# Engineering a Novel Multifunctional Green Fluorescent Protein Tag for a Wide Variety of Protein Research

**DOI:** 10.1371/journal.pone.0003822

**Published:** 2008-12-02

**Authors:** Takuya Kobayashi, Nobuhiro Morone, Taku Kashiyama, Hideto Oyamada, Nagomi Kurebayashi, Takashi Murayama

**Affiliations:** 1 Department of Pharmacology, Juntendo University School of Medicine, Bunkyo-ku, Tokyo, Japan; 2 Department of Ultrastructural Research, National Institute of Neuroscience, National Center of Neurology and Psychiatry, Kodaira, Tokyo, Japan; 3 Department of Pharmacology, School of Medicine, Showa University, Shinagawa-ku, Tokyo, Japan; University of Munich and Center of Integrated Protein Science, Germany

## Abstract

**Background:**

Genetically encoded tag is a powerful tool for protein research. Various kinds of tags have been developed: fluorescent proteins for live-cell imaging, affinity tags for protein isolation, and epitope tags for immunological detections. One of the major problems concerning the protein tagging is that many constructs with different tags have to be made for different applications, which is time- and resource-consuming.

**Methodology/Principal Findings:**

Here we report a novel multifunctional green fluorescent protein (mfGFP) tag which was engineered by inserting multiple peptide tags, i.e., octa-histidine (8×His), streptavidin-binding peptide (SBP), and c-Myc tag, in tandem into a loop of GFP. When fused to various proteins, mfGFP monitored their localization in living cells. Streptavidin agarose column chromatography with the SBP tag successfully isolated the protein complexes in a native form with a high purity. Tandem affinity purification (TAP) with 8×His and SBP tags in mfGFP further purified the protein complexes. mfGFP was clearly detected by c-Myc-specific antibody both in immunofluorescence and immuno-electron microscopy (EM). These findings indicate that mfGFP works well as a multifunctional tag in mammalian cells. The tag insertion was also successful in other fluorescent protein, mCherry.

**Conclusions and Significance:**

The multifunctional fluorescent protein tag is a useful tool for a wide variety of protein research, and may have the advantage over other multiple tag systems in its higher expandability and compatibility with existing and future tag technologies.

## Introduction

Protein complexes mediate the majority of cellular processes. Information on localization, structure, and interaction of such protein complexes provides key insights into their functions. Genetically encoded protein tags are a useful tool for characterization of protein complexes. There are a variety of tags for localizing proteins in living cells. Among them, the most widely used are *Aequorea victoria* green fluorescent protein (GFP) and the related fluorescent proteins, which are easily fused to proteins of interest [1], [2]. Because the N- and C-termini of GFP are closely located to each other, this protein can be inserted in the middle of the fusion partner, as well as fusion at its N- and C-termini [3]. This is great advantage in case that the protein has intolerant of tag fusion at both N- and C-termini.

Structural and interaction analysis of the protein complexes requires isolation of the complex. A variety of affinity tags have been developed, which strongly bind to a specified ligand [4], [5]. The use of multiple tags, such as tandem affinity purification (TAP) strategy, has recently become popular for purification of the protein complexes with a high purity [6]. Epitope tags, small peptides to which commercially available highly specific antibody bind, are used for a wide variety of immunological detections [7]. Epitope tags are also useful for affinity isolation of the protein complexes by immunoprecipitation.

In general, cellular localization and isolation of protein complexes are determined using fusion proteins with different tags; GFP-tagged protein for localization and affinity tagged-protein for structural and protein composition analysis. However, making of different constructs is time- and resource-consuming, especially in case of analyzing a large number of proteins, e.g., genome-wide analysis. In addition, it is better to correlate the two determinations using the same fusion protein. Development of a single tag with multifunction is therefore highly desirable.

**Figure 1:**
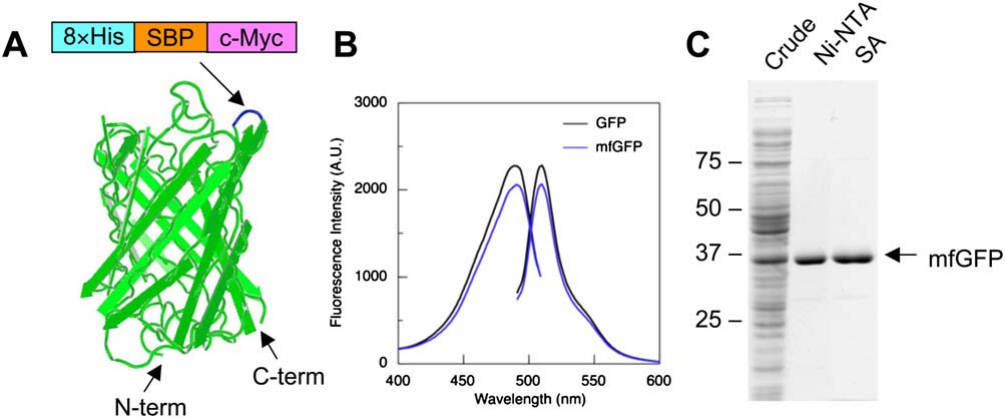
Design and characterization of multifunctional GFP (mfGFP). (A) Schematic representation of mfGFP. Octa-histidine (8×His), streptavidin-binding peptide (SBP), and c-Myc tag were inserted in tandem after Asp173 within a loop between the β-strands (blue) that is located on the opposite side of N- and C-termini. (B) Excitation and emission spectra of wild type GFP (with N-terminal His-tag) and mfGFP that were expressed in *E. coli* and purified by nickel-nitrilotriacetic acid (Ni-NTA) beads. (C) Purification of mfGFP. mfGFP was purified by either Ni-NTA beads or streptavidin beads, processed on an SDS-polyacrylamide gel, and stained with CBB.

GFP forms a rigid and stable 11-stranded β-barrel structures [1], [2] (see Fig. 1A). It has been shown that GFP is tolerant to insertion of foreign peptides within certain loops between the β-strands [8] and thus can be a scaffold for short peptides [9], [10]. In this study, we engineered “multifunctional GFP” (mfGFP), in which the multiple peptide tags (affinity tags and epitope tags) were inserted in tandem into a loop of GFP. The mfGFP successfully monitored localization of the fusion protein in living cells, isolated the protein complex in native form with a high purity, and detected the protein in immunofluorescence and immuno-electron microscopy (EM). mfGFP is a useful tool for a wide variety of protein research including live-cell imaging, affinity isolation, and immunological detection.

## Results and Discussion

The mfGFP was engineered by inserting three tags (a total of 67 residues including linkers) into GFP in tandem: octa-histidine (8×His) and streptavidin-binding peptide (SBP) for affinity isolation, and c-Myc for immunological detection (see Supplementary Figure S1). The Asp173-Gly174 was chosen as an insertion site, because it is located at the opposite side of N- and C-termini that would allow tags to work effectively by minimizing steric hindrance provided by the fusion partner (Fig. 1A).

We initially tested the properties of mfGFP which was expressed in bacterial system. MfGFP was expressed in *E. coli* as a soluble protein with bright green fluorescence. Excitation and emission spectra of the mfGFP were almost identical with those of wild type GFP with only a slight reduction of peak fluorescent intensity (Fig. 1B). Thus, insertion of the tags did not substantially affect the fluorescence properties of GFP. MfGFP was isolated as a single band in SDS-PAGE by nickel-nitrilotriacetic (Ni-NTA) acid and streptavidin (SA) chromatography (Fig. 1C). These findings indicate that 8×His and SBP work well in the loop of mfGFP.

**Figure 2:**
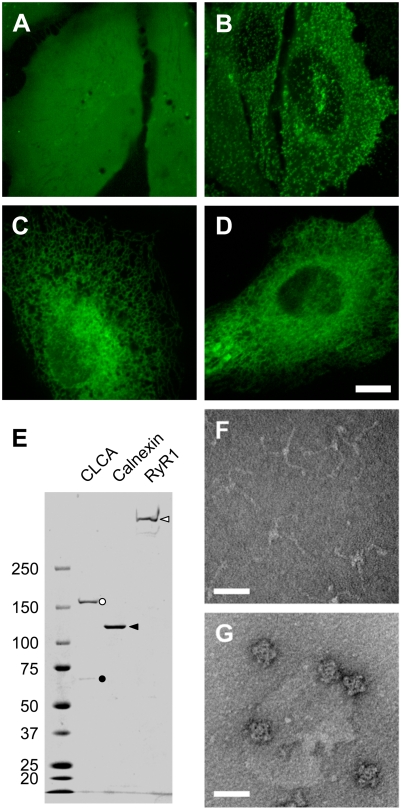
Localization of mfGFP fusion proteins in living cells and isolation of mfGFP-fusion protein complexes. (A–D) HeLa cells were transfected with expression vectors of either mfGFP alone (A) or mfGFP fused with clathrin light chain A (CLCA) (B), calnexin (C), and type 1 ryanodine receptor (RyR1) (D). Whereas mfGFP alone distributed throughout the cells, fusion proteins were localized at the expected site. Scale bars, 10 µm. (E) mfGFP-fusion protein complexes were isolated by streptavidin column chromatography using SBP-tag. The isolated fractions were processed on an SDS-polyacrylamide gel, and stained with CBB. CLCA (black circle) co-purified clathrin heavy chain (CHC) (white circle). Calnexin and RyR1 were isolated as a single band. (F and G) Negative staining EM observation of isolated CLCA and RyR1 fractions. CLCA exhibited three-armed pinwheel morphology of triskelion (F), whereas RyR1 exhibited characteristic quarterfoil appearance (G). Scale bar, 50 nm.

Next, we tested the ability of mfGFP in mammalian cells. HeLa cells expressing mfGFP alone demonstrated a homogeneous fluorescence throughout the cells, being consistent with the distribution of wild type GFP (Fig. 2A). This suggests that mfGFP is not associated with any specific organelle or cellular structures. mfGFP was fused to several membrane proteins to evaluate its fusion properties: clathrin light chain A (CLCA), a 30 kDa component of clathrin coated pits [11] (Fig. 2B), calnexin, a 90 kDa endoplasmic reticulum (ER) chaperon protein [12] (Fig. 2C), and ryanodine receptor type 1 (RyR1), a large homotetrameric Ca^2+^ release channel protein in the ER [13] (Fig. 2D). The mfGFP was fused at C-terminus for CLCA and calnexin or in the middle of the coding sequence for RyR1 [13]. All the fusion proteins demonstrated appropriate cellular localization, indicating that mfGFP can monitor localization of the fusion partner as is the case with wild type GFP.

The mfGFP-tagged protein complexes were isolated by SA column using SBP-tag (see Materials and Methods). SBP-tag is superior than His-tag in purity of the isolated proteins in higher organisms including mammalian cells [14]. The isolated CLCA fraction contained two polypeptide bands (∼160 and ∼60 kDa) which correspond to clathrin heavy chain (CHC) and CLCA-mfGFP, respectively (Fig. 2E). CLCA interacts with CHC to form “triskelion” which is an assemble unit of clathrin cage [11]. Negative staining EM of the isolated CLCA fraction exhibited characteristic three-armed pinwheel morphology of triskelion (Fig. 2F). Calnexin fraction exhibited a major 120 kDa polypeptide of calnexin-mfGFP (Fig. 2E). RyR1 was successfully isolated as a single high molecular weight band in SDS-PAGE (Fig. 2E). A quarterfoil appearance in negative staining EM (Fig. 2G) together with a specific [^3^H]ryanodine binding (data not shown) confirmed that the isolated RyR1 forms functional Ca^2+^ release channel [13]. Thus, mfGFP can isolate protein complexes in a native form, at both terminal and internal fusion.

**Figure 3:**
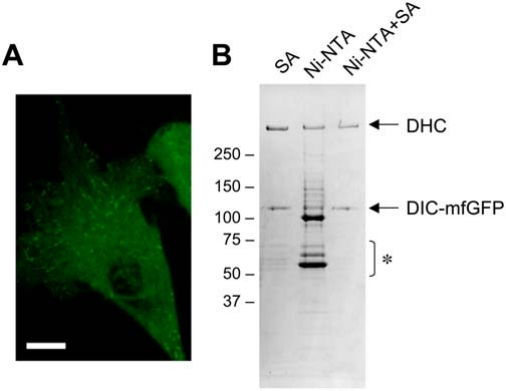
Tandem Affinity Purification (TAP) of cytoplasmic dynein complexes using His-tag and SBP-tag. mfGFP was fused to C-terminus of dynein intermediate chain (DIC). (A) Localization of DIC-mfGFP in living HeLa cells. Scale bar, 10 µm. (B) Isolation of cytoplasmic dynein complexes. Streptavidin (SA) column chromatography isolated DIC-mfGFP and dynein heavy chain (DHC) with some contaminating proteins in 50–75 kDa range (asterisk). Ni-NTA chromatography isolated the two proteins, but many contaminants were also seen. TAP strategy with Ni-NTA and SA (Ni-NTA + SA) isolated the two proteins with a significantly reduction of contaminants in 50–75 kDa range.

Tandem affinity purification (TAP) is a powerful strategy for protein interaction analysis, which isolates the protein complex with a high purity using two distinct affinity tags [6]. We tested 8×His tag and SBP-tag inserted into mfGFP as TAP tags using 74-kDa dynein intermediate chain (DIC). DIC interacts with ∼500 kDa dynein heavy chain (DHC) to form the cytoplasmic dynein complex. mfGFP was fused to C-terminus of DIC. When expressed in HeLa cells, DIC-mfGFP exhibited as dots in the cytoplasm which move along with microtubules (Fig. 3A). SA column chromatography isolated two major bands; >250 kDa for DHC and ∼110 kDa for DIC-mfGFP (Fig. 3B). Some contaminants were also detected in 50–75 kDa range. Ni-NTA chromatography with 8×His tag isolated the two bands but many contaminants were also detected. This indicates that 8×His tag alone is insufficient to isolate the protein complexes from the mammalian cells. The SA chromatography of the eluted fraction from Ni-NTA, however, significantly reduced contaminants in 50–75 kDa range relative to the SA chromatography alone. These findings suggest that 8×His tag and SBP-tag work well as TAP tags. Several peptide affinity tags for TAP strategy have been proposed, which include calmodulin-binding peptide, StrepTagII, and FLAG tag [6], [15]. These tags would also be good candidates for TAP strategy with mfGFP.

**Figure 4:**
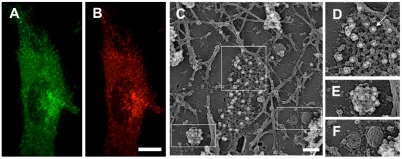
Immunofluorescence and immuno-EM of CLCA-mfGFP expressing cells with c-Myc-specific antibody. (A, B) Immunofluorescence microscopy. The cells were fixed and stained with c-Myc-specific antibody followed by AlexaFluor594 labeled rabbit IgG-specific secondary antibody. The AlexaFluor594 fluorescence (B) was overlapped with GFP fluorescence (A). Scale bar, 10 µm. (C–F) Frozen-replica immuno-EM using 10 nm colloidal gold. Boxed areas in (C) are shown at higher magnification (D–F). Gold particles (white dots, marked by an arrow) were detected on the clathrin coated pits (D) and vesicles (E), but not caveolae (F) or actin cytoskeleton (C). Scale bar, 100 nm.

Properties of the epitope tag in immunological detections were tested with CLCA. GFP fluorescence of HeLa cells stably expressing CLCA-mfGFP distributed in a punctate pattern within the cell (Fig. 4A). AlexaFluor594 staining with c-Myc-specific antibody co-localized with GFP fluorescence (Fig. 4B). In frozen-replica immuno-EM [16], clathrin coated pits and vesicles were observed in the cytoplasmic surface of the plasma membrane together with caveolae and membrane cytoskeletons formed by actin filaments (Fig. 4C). c-Myc tag was detected by c-Myc-specific antibody and 10 nm colloidal gold-conjugated secondary antibody. Gold label was specifically detected on clathrin coated pits and vesicles (Fig. 4D,E), but not on caveolae or actin cytoskeletons (Fig. 4C,F). These findings demonstrated that c-Myc tag in mfGFP works well both in immunofluorescence and immuno-EM studies.

A pair of fluorescent proteins (e.g., CFP and YFP) is widely used for fluorescence resonance energy transfer (FRET), a strategy for interaction analysis of two proteins [17]. A detailed cellular localization by immuno-EM and direct interaction by immunoprecipitation of the two proteins may further support the FRET results. However, no antibodies are so far available, which can distinguish fluorescent proteins from the same origins (e.g., CFP, GFP, and YFP). Fluorescent proteins with different epitope tags can be easily distinguished by commercially available tag-specific antibodies. Insertion of epitope tags into fluorescent proteins is highly useful for immunological detections of multiple fluorescent proteins.

The fluorescent proteins so far discovered share typical 11-stranded β-barrel structures [1], [2]. The tag insertion will be applicable to the other fluorescent proteins. We successfully inserted several tags into the corresponding loop of mCherry, a red fluorescent protein derived from the *Discosoma* sp. [18] (supplementary Figure S2). In addition, because the loop of GFP is enough tolerant to insertion of the large foreign peptides [8], the tags can also be varied depending on applications. Suitable combinations of fluorescent proteins and inserted tags will be adjusted for individual proteins and/or research applications.

Linking GFP and affinity tag in tandem is used as a simple approach for live-cell imaging and protein isolation [19]. When the tag is inserted into the coding sequence of the protein of interest, it would be expected that affinity tag sometimes does not work due to steric hindrance produced by GFP and the protein of interest that surround the affinity tag. In contrast, the affinity tag in mfGFP work well irrespective of the insertion site, because the affinity tag is located at the opposite side of N- and C-termini of GFP that are linked to the protein of interest (see Fig. 1A). Transposon-based random insertion of GFP is an interesting approach to study structure-function relationship of the relatively large protein [3]. mfGFP will also be useful for the strategy.

The engineered tag systems for both live-cell imaging and protein isolation have recently been available (HaloTag and SNAP-tag) [20], [21]. These tags are derived from some enzymes and covalently attach the specific ligand to themselves. By choosing the ligand, they can be used for live-cell imaging (with fluorescent ligands) or protein isolation (with affinity ligands such as biotin). As compared with these tags, the main advantage of mfGFP is its high expandability and compatibility with existing and future tag technologies. Whereas HaloTag and SNAP-tag systems are limited by its ligand design, all the peptide tags are a potential candidate for mfGFP. In addition, easy-to-use format is of great advantage of mfGFP. GFP and the related fluorescent proteins are widely used in the world and the system works just by replacing the original GFP by mfGFP. Thus, mfGFP is superior to the other tag systems. mfGFP will be a powerful tool for a wide variety of protein research, especially in analyzing a number of target proteins, such as genome-wide analysis of unknown proteins.

## Materials and Methods

### Construction of mfGFP and fusion proteins

The gene encoding the GFP was PCR amplified from pEGFP-N1 (Clontech) and subcloned into the pCold III vector (Takara). The DNA sequence encoding octa-histidine (8×His), streptavidin-binding peptide (SBP), and c-Myc tag were inserted after Asp173 by several steps of PCR. The DNA sequence of the product was confirmed by DNA sequencing.

cDNAs encoding CLCA, calnexin, and DIC were obtained from human embryonic kidney (HEK) cells by RT-PCR and ligated into the pcDNA5/FRT/TO expression vector (Invitrogen). cDNA encoding rabbit RyR1 was constructed as described elsewhere [22]. The mfGFP was fused either at the C-terminus (CLCA, calnexin, and DIC), or in the middle of the coding sequence (RyR1, after Ala1397). For generation of stable transfectants, cDNA fragments for fusion proteins were ligated into pIREShyg2 vector (Clontech). A detailed procedure of construction (with sequences of PCR primers) and full amino acid sequence of mfGFP are shown in supporting information (Supplementary Methods S1 and Supplementary Figure S1).

### Fluorescent measurements

Wild type GFP (with N-terminal hexa-histidine tag) and mfGFP were expressed in *E. coli*. The bacterial cell pellet was lysed by sonication and proteins were purified by Profinity IMAC Ni-charged resin (Bio-rad). The protein amount was measured using the Advanced protein assay reagent (Cytoskeleton Inc.) with bovine serum albumin as a standard. Fluorescence spectra of wild type GFP and mfGFP were measured in a Hitachi F-4500 fluorescence spectrophotometer in a buffer containing 150 mM NaCl and 20 mM sodium phosphate, pH 7.2.

### Cell culture

HeLa cells were grown in Dulbecco's modified eagle medium (DMEM) supplemented with 10% fetal calf serum, 2 mM glutamine, and antibiotics (penicillin/streptomycin). Transfection of the expression vector was carried out using Lipofectamine LTX reagent (Invitrogen). Stable transfectants were generated by selecting colonies resistant to hygromycin after transfection of pIREShyg2 expression vector. For isolation of protein complexes, stable and inducible HEK cell lines were generated using Flp-In T-REx system according to the manufacturer's instructions.

### Fluorescence microscopy

HeLa cells were grown on the collagen-coated coverslips. Fluorescence imaging was carried out with a confocal laser scanning microscope system (Yokogawa, CSU22) equipped with an Argon Krypton Ion Confocal Laser System (488 and 568 nm excitation). For immunofluorescent microscopy, anti-c-Myc antibody (Abcam, ab9106) and AlexaFluor594-labeled goat anti-rabbit IgG (Invitrogen, A11012) were used.

### Isolation of mfGFP-tagged protein complexes

For CLCA, microsomes containing clathrin coated vesicles were prepared from HeLa cells stably expressing CLCA-mfGFP according to Prasad et al [23]. Clathrin triskelions were extracted with high ionic strength solution [23] and applied onto a HiTrap streptavidin column (GE Healthcare) that had been equilibrated with buffer A (0.5 M NaCl, 20 mM MOPS, pH 7.4, 0.3 M sucrose, and 2 mM dithiothreitol). The column was washed with the buffer and the bound protein was eluted by buffer A containing 2.5 mM d-desthiobiotin. For calnexin and RyR1, microsomes prepared from the HEK cells were solubilized with buffer A containing 1% CHAPS and 0.5% soybean phosphatidylcholine, and the HiTrap streptavidin column chromatography was carried out with buffer A containing 0.5% CHAPS.

### Tandem affinity purification of dynein complex

HEK cells expressing DIC-mfGFP were homogenized with buffer B (0.2 M NaCl, 50 mM Tris-HCl, pH 7.5, 10% sucrose, 5 mM MgCl2, 0.1 mM ATP, and 0.1 mM dithiothreitol) containing 0.05% Triton X-100, and the supernatant after ultracentrifugation was incubated with Profinity IMAC Ni-charged resin in buffer B containing 20 mM imidazole. After washing with the buffer, the bound protein was eluted with buffer B containing 300 mM imidazole. The eluted fraction was then applied onto a HiTrap streptavidin column that had been equilibrated with buffer C (0.2 M NaCl, 50 mM Tris-HCl, pH 7.5, 10% sucrose, 5 mM MgCl2, 0.1 mM ATP, and 0.5 mM dithiothreitol). After extensive washing, the bound protein was eluted with buffer C containing 2.5 mM d-desthiobiotin.

### Negative staining EM

Negative staining of the clathrin triskelion and RyR1 was carried out according to the standard procedures using 2% uranyl acetate. The specimens were examined in a Hitachi H-7100 electron microscope operating at 75 kV.

### Freeze-Etch EM of the Cytoplasmic Cell Surface

Rapid-freeze, deep-etch EM for the cytoplasmic surface of the plasma membrane was carried out according to previously described methods [16], [24], [25]. HeLa cells cultured on glass coverslips were unroofed from the apical cell membrane and fixed. After being quenched and blocked, the cells were treated with anti-c-Myc antibody (Abcam, ab9106) followed by 10 nm gold-conjugated anti-rabbit IgG secondary antibody (GE Healthcare). Specimens were rapidly frozen to contact with the pure copper block cooled by liquid helium by using the rapid-freezing device (Polaron, USA) and the frozen cytoplasmic surface was deeply etched and rotary shadowed with platinum/carbon and carbon, using the freeze-etching device (Bal-Tec BAF060, Liechtenstein). The replica was observed by TEM (FEI Tecnai *Sphera* or *Sprit*, USA).

## Supporting Information

Supplementary Methods S1 (0.03 MB DOC)

Figure S1 Amino acid sequence of mfGFP. Peptide tags inserted between Asp173 and Gly174 (boxed) are shown in color: 8×His in light blue, streptavidin-binding peptide (SBP) in yellow, c-Myc tag in green, and linkers in grey.
(0.35 MB TIF)

Figure S2 Design and characterization of multifunctional mCherry. (A) Schematic representation of multifunctional mCherry. Octa-histidine tag (8×His) and hemagglutinin (HA) tag were inserted in tandem after Asp174 within a loop between the β-strands (yellow) that is located on the opposite side of N- and C-termini. (B) Live-cell imaging of clathrin light chain A (CLCA)-multifunctional mCherry. Scale bar, 10 µm. (C) Detection of HA tag in immunofluorescent microscopy. The cells were fixed and stained with anti-HA antibody followed by AlexaFluor488 labeled anti-mouse IgG secondary antibody. Left, mCherry fluorescence; Right, AlexaFluor488 fluorescence. Scale bar, 10 µm. (D) Detection of HA tag in frozen-replica immuno-electron microscopy using 10 nm colloidal gold. Gold particles (white dots) were detected on the clathrin coated pits and vesicles. Scale bar, 100 nm.
(1.92 MB TIF)
