# Supplementary material for: Engineering a Novel Multifunctional Green Fluorescent Protein Tag for a Wide Variety of Protein Research
Source: PLoS One. 2008 Dec 2;3(12):e3822. doi: 10.1371/journal.pone.0003822 (PMC2585475; doi:10.1371/journal.pone.0003822)
Supplement: Supplementary Methods S1 — (0.03 MB DOC) [file pone.0003822.s001.doc]

**Supplementary Methods**

Construction of mfGFP

The gene encoding the GFP was amplified from pEGFP-N1 (Clontech) using a pair of PCR with Sac I and Xho I restriction sites on 5’ and 3’ ends, respectively (underlined): 5'-GGGGAGCTCGGATCCGGAGTGAGCAAGGGC-3' and 5'-GGGCTCGAGTTCAGATCCGGACCCCTTGTA-3', and subcloned into the pCold III vector (Takara). The DNA sequence GSGHHHHHHHHGS encoding octa-histidine with linkers was inserted after Asp173 by inverse PCR using a pair of primers: 5'-TGGTGATGGTGTCCAGAGCCGTCCTCGATGTTGTGGCGGATC-3' and 5'-TCACCATCACCATGGCTCCGGCAGCGTGCAGCTCGCCGACC-3' (underlines correspond to octa-histidine sequence). The DNA sequence encoding streptavidin-binding peptide (SBP)-tag was amplified from pT7-SBP-2 (Sigma) using a pair of PCR primers with Nco I sites on both ends (underlined): 5'-GGGCACCATGGCTCTATGGACGAAAAAACCACCGGT-3' and 5'-GGAGCCATGGGAGGGTTCACGCTGACCCTGCGG-3', and subcloned into a Nco I site after the His-tag. The DNA sequence GSGEQKLISEEDLGSG encoding c-Myc (underlined) with GS linkers was inserted after the SBP-tag by inverse PCR using a pair of primers: 5'-TCTGAAGAGGATCTGGGCTCCGGCAGCGTGCAGCTC-3' and 5'-GATGAGTTTCTGCTCGGATCCATGGGAGGGTTCACGCTGACC-3'. The DNA sequence of the product was confirmed by DNA sequencing.

Construction of mfGFP fusion proteins

A cDNA encoding clathrin light chain A (CLCA) and calnexin was obtained from human embryonic kidney (HEK) cells by RT-PCR using following primer pairs (underlines represent the target sequence for restriction enzymes): 5'-AGGCTAGCCACCATGGAAGGTAGGCATATGGAGCTC-3' and 5'-CCGGTACCGTGCACCAGCGGGGCCTG-3' for CLCA and 5'-CCGCTAGCCACCATGGAAGGGAAGTGGTTGCTG-3' and 5'-CCGGTACCCTCTCTTCGTGGCTTTCTGTT-3' for calnexin. The PCR product was ligated into the pcDNA5/FRT/TO expression vector (Invitrogen). MfGFP was fused to the C-terminus. For generation of stable transformants, cDNA fragments for fusion proteins were ligated into pIREShyg2 vector (Clontech).

For inserting the mfGFP into the D2 region of RyR1, adaptor sequences encoding the GS (x 3) linkers with the recognition sites of the restriction enzyme Sfi I were generated in frame to both ends of the mfGFP cDNA by PCR sequentially. The first PCR for creating the GS (x3) linkers at both ends of mfGFP was performed from the template of mfGFP cDNA with primer pairs of 5'-GGAATTCGGCGGAGGCGGGGGTAGCGGGGGTGGATCTGGAGGGGGCTCCGTGAGCAAGGGCGAGGAGCTG-3' and 5'-GGAATTCTCCGCCACTGCCACCCCCCGAGCCACCTCCTGACCCGCCACCCTTGTACAGCTCGTCCATGCC-3'. The second PCR for addition of the Sfi I sites to both ends of the GS (x3) - linkered mfGFP cDNA was carried out on the template of the first PCR products with primer pairs (Sfi I sites were underlined) of 5’-CCGGCCAAGAAGGCCGGCGGGGGTAGCGGGGGTGGATCTGGAGGG-3’ and 5’-CAGGCCATCTTGGCCCCACTGCCACCCCCCGAGCCACCTCCTGAC-3’. Final PCR products were subcloned into pTA2 vector by the TA-cloning method according to manufacturer’s instructions (TOYOBO Inc., JAPAN). After the Sfi I digestion of the GS-linkered mfGFP cloned pTA2 vector, the cDNA fragments of GS linkered mfGFP with Sfi I cutting ends were inserted into the Sfi I site of the cDNA cassette 4 of RyR1 (BS-RyR1cs4 referred in 22). The BS-RyR1cs4 including the GS linkered mfGFP cDNA were cut by Bst BI / Mlu I and subcloned back to into its original site of the full length RyR1cDNA (pBS-RyR1c referred in 22). The full length RyR1 cDNA containing the GS-linkered mfGFP cDNA at the position of cDNA encoding the D2 region of RyR1 was cut by Xba I / Eco RV and subcloned into the multiple cloning sites (MSC) of pcDNA/FRT/TO vector (Invitrogen) modified by addtion of the Nhe I site in the MSC.
