# Supplementary figures and images for: Engineering a Novel Multifunctional Green Fluorescent Protein Tag for a Wide Variety of Protein Research
Source: PLoS One. 2008 Dec 2;3(12):e3822. doi: 10.1371/journal.pone.0003822 (PMC2585475; doi:10.1371/journal.pone.0003822)

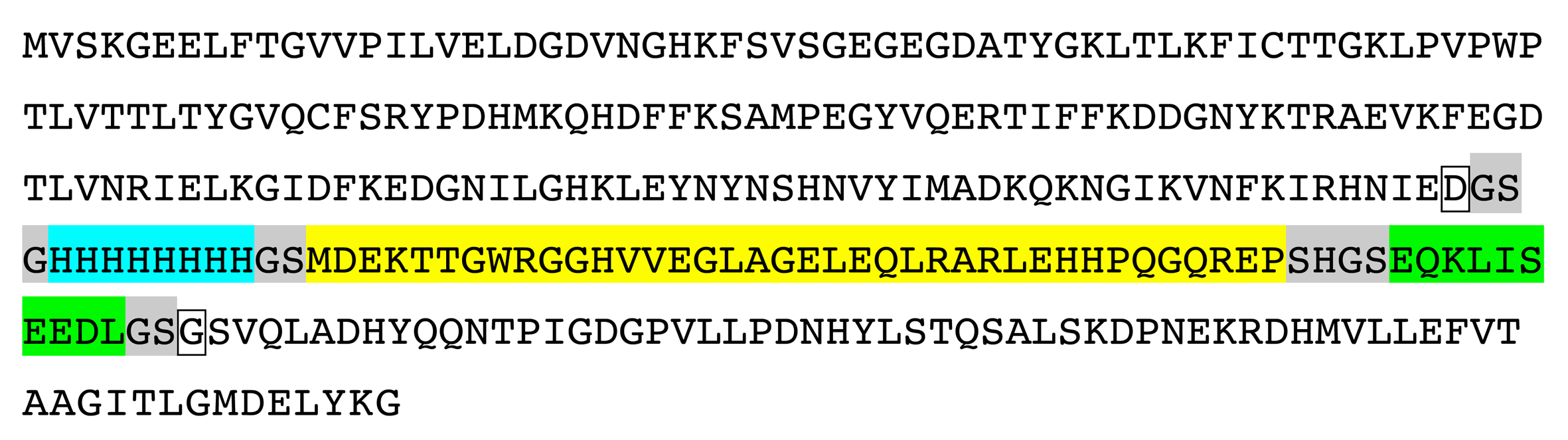

Supplement: Figure S1 — Amino acid sequence of mfGFP. Peptide tags inserted between Asp173 and Gly174 (boxed) are shown in color: 8×His in light blue, streptavidin-binding peptide (SBP) in yellow, c-Myc tag in green, and linkers in grey. (0.35 MB TIF) [file pone.0003822.s002.tif]

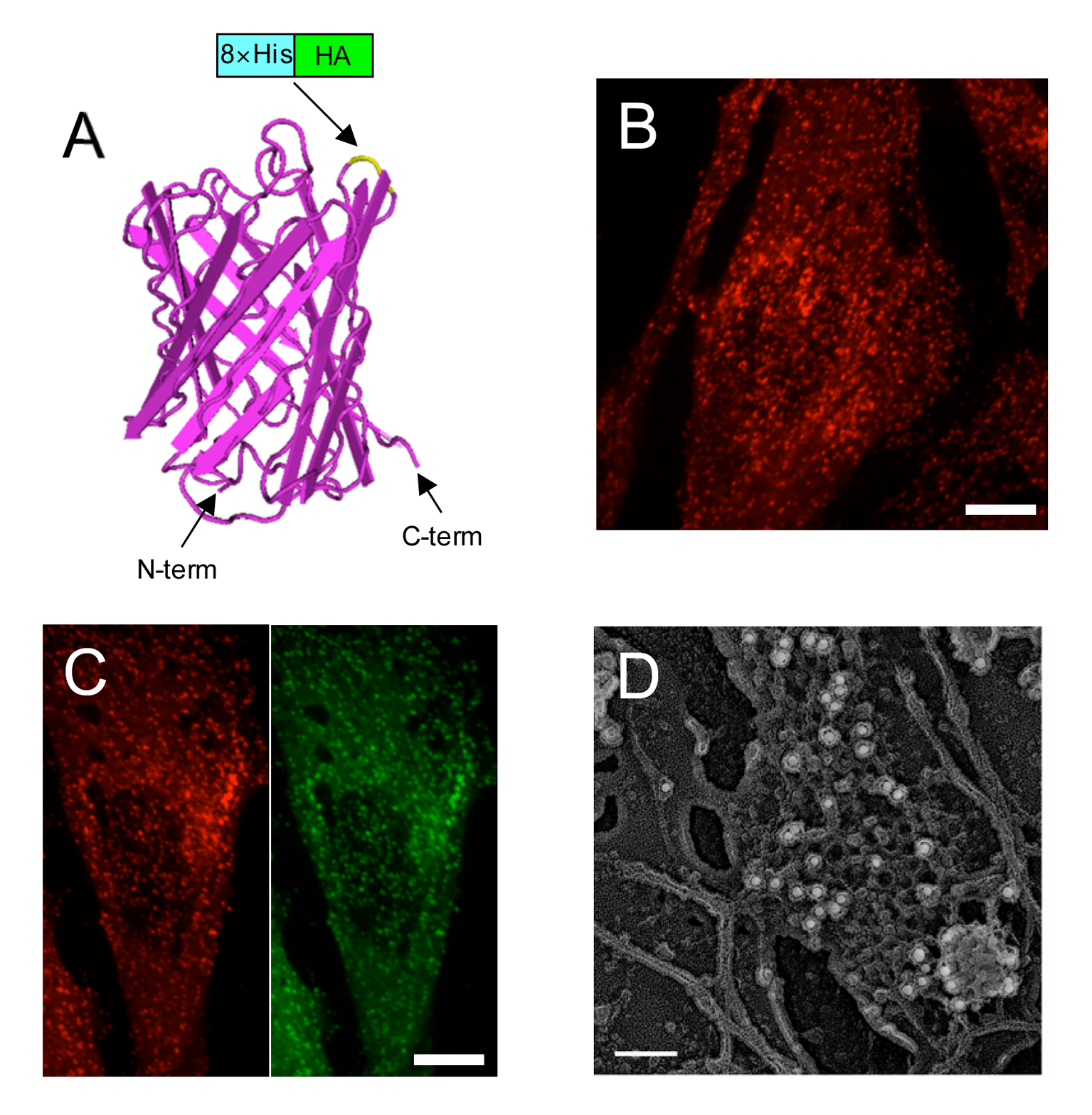

Supplement: Figure S2 — Design and characterization of multifunctional mCherry. (A) Schematic representation of multifunctional mCherry. Octa-histidine tag (8×His) and hemagglutinin (HA) tag were inserted in tandem after Asp174 within a loop between the β-strands (yellow) that is located on the opposite side of N- and C-termini. (B) Live-cell imaging of clathrin light chain A (CLCA)-multifunctional mCherry. Scale bar, 10 µm. (C) Detection of HA tag in immunofluorescent microscopy. The cells were fixed and stained with anti-HA antibody followed by AlexaFluor488 labeled anti-mouse IgG secondary antibody. Left, mCherry fluorescence; Right, AlexaFluor488 fluorescence. Scale bar, 10 µm. (D) Detection of HA tag in frozen-replica immuno-electron microscopy using 10 nm colloidal gold. Gold particles (white dots) were detected on the clathrin coated pits and vesicles. Scale bar, 100 nm. (1.92 MB TIF) [file pone.0003822.s003.tif]
